# Cation Functionalization Alters Cohesion, Not Interaction Type, in Ionic Liquids: Evidence from Infinite Dilution Activity Coefficients

**DOI:** 10.1021/acs.jpcb.6c02483

**Published:** 2026-06-01

**Authors:** Kamil Paduszyński, Marta Królikowska

**Affiliations:** Department of Physical Chemistry, 49566 Faculty of Chemistry Warsaw University of Technology, Noakowskiego 3, 00-664 Warsaw, Poland

## Abstract

The effect of cation functionalization on solute–solvent interactions in ionic liquids (ILs) was investigated through infinite dilution activity coefficients (IDACs) of 34 molecular solutes in 1-ethyl-1-methylpyrrolidinium diethyl phosphate and 4-ethyl-4-methylmorpholinium diethyl phosphate. New experimental IDAC data were obtained by gas–liquid chromatography over the temperature range from (308.15 to 358.15) K. The results show that insertion of an oxygen atom into the saturated cation ring systematically increases IDACs and thus reduces solvent affinity, with the effect being strongest for nonpolar solutes and progressively smaller for polar compounds. This behavior indicates that cation functionalization primarily raises the baseline thermodynamic cost of solvation rather than altering the fundamental hierarchy of solute–IL interactions. For both ILs, nonpolar solvation is dominated by the free-energy cost of cavity formation and is both enthalpically and entropically unfavorable, whereas polar solutes are stabilized mainly by favorable dipolar and hydrogen-bonding interactions. Temperature-dependent analysis further shows that the cation substitution modifies both enthalpic and entropic contributions to solvation while preserving the overall interaction pattern. Linear solvation energy relationship analysis, regular-solution treatment, and COSMO-RS calculations support this interpretation, showing that the two ILs differ mainly in overall solvent affinity rather than in interaction type. Overall, the results identify cation functionalization as a rational means of tuning solvation thermodynamics in ILs through controlled modification of the liquid-state interaction balance.

## Graphical abstract

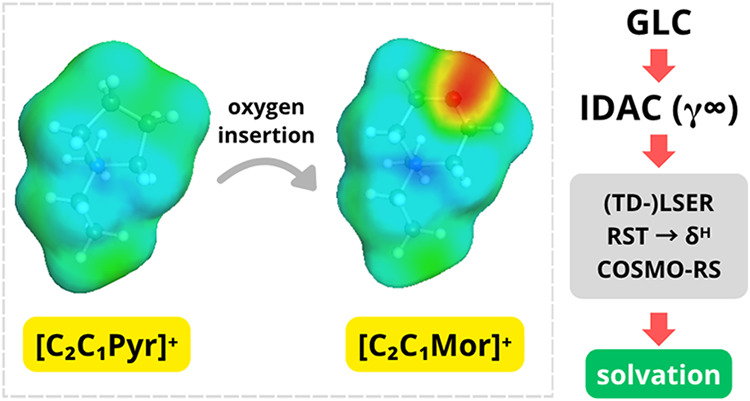

## Introduction

Ionic liquids (ILs) are salts composed entirely of ions that are liquid at or near ambient temperature, whose physicochemical behavior arises from a nontrivial balance of Coulombic, hydrogen-bonding, dispersion, and steric interactions.^1,2^ Their defining characteristics include negligible vapor pressure and high thermal stability,^3^ as well as intrinsic structural heterogeneity associated with the coexistence of polar and nonpolar nanodomains.^4,5^ These features distinguish ILs fundamentally from conventional molecular solvents and make them attractive model systems for probing the role of competing intermolecular interactions in governing thermodynamic and transport properties.^6^ Moreover, the modular nature of ILs, which allows independent variation of cation and anion structures, enables systematic tuning of their interaction capabilities and provides a powerful framework for investigating structure–property relationships in complex liquids.^7,8^

Understanding molecular interactions in ILs is essential for both fundamental insight and rational chemical design. Solute–IL interactions determine key macroscopic properties such as solubility, selectivity, and phase equilibria, which are directly relevant to applications in separation processes, extraction, and reaction media design.^9,10^ These interactions can be investigated using a range of experimental and theoretical approaches, among which thermodynamic measurements play a central role. In particular, infinite dilution activity coefficients (IDACs) provide direct thermodynamic measures that are highly sensitive to solute–IL interactions in the limit of vanishing solute concentration, where solute–solute effects are eliminated.^11^ As such, IDACs serve as a robust basis for quantifying interaction strengths and identifying dominant contributions to solvation,^12^ as well as for parametrizing predictive models.^13^

Over the past two decades, systematic studies of IDACs in ILs have been conducted by numerous research groups, leading to extensive data sets covering a wide variety of IL families and molecular solutes.^14^ Among these, pyrrolidinium-based ILs have been relatively well characterized across a broad range of anions, including bis­(trifluoromethylsulfonyl)­imide [NTf_2_], trifluoromethanesulfonate [OTf], dicyanamide [DCA], tricyanomethanide [TCM], tetracyanoborate [TCB], thiocyanate [SCN], and others.^15–26^ These studies often include extensive solute sets and temperature-dependent data, enabling detailed thermodynamic analyses and systematic evaluation of both cation alkyl chain length effects and anion-dependent interaction patterns. In selected cases, functionalized pyrrolidinium cations containing ether groups have also been examined.^19,21^

In contrast, morpholinium-based ILs remain significantly less explored, with available IDAC studies limited to a relatively small number of systems and predominantly focused on fluorinated anions such as [NTf_2_], [TCM], [TCB], and [FAP].^27–31^ Although these works indicate that incorporation of an oxygen atom into the cation ring increases polarity and modifies hydrogen-bonding characteristics, the available data set remains insufficient to establish general structure–property relationships or to disentangle cation-specific effects from those imposed by the anion.

As a result, direct comparisons between pyrrolidinium and morpholinium cation families are scarce, and the role of the ring oxygen atom in modulating solute–IL interactions cannot be unambiguously assessed. This limitation is particularly evident for ILs containing strongly coordinating or basic anions. While pyrrolidinium-based systems incorporating phosphate-type anions have been investigated in the context of IDAC measurements,^32^ no analogous data are currently available for morpholinium counterparts. Consequently, the combined effect of cation functionalization and anion-specific interactions remains unresolved.

In this work, we investigate the effect of cation functionalization on solute–IL interactions by comparing IDACs of a diverse set of molecular solutes in two closely related ILs, 1-ethyl-1-methylpyrrolidinium diethyl phosphate and 4-ethyl-4-methylmorpholinium diethyl phosphate; the chemical structures of the investigated ions are shown in Figure 1. The introduction of an oxygen atom into the saturated cation ring provides a well-defined structural modification that alters polarity, hydrogen-bonding characteristics, and conformational flexibility. The central question addressed here is whether cation functionalization primarily alters specific solute–IL interactions or instead modifies the baseline cohesive properties of the liquid, thereby uniformly shifting solvation thermodynamics. To address this question, new experimental IDAC data are reported over a range of temperatures and analyzed to elucidate trends in solvation behavior. The experimental results are further interpreted using linear solvation energy relationships, regular solution theory, and COSMO-RS modeling to separate dispersive, polar, and hydrogen-bonding contributions to solvation.

**Figure 1:**
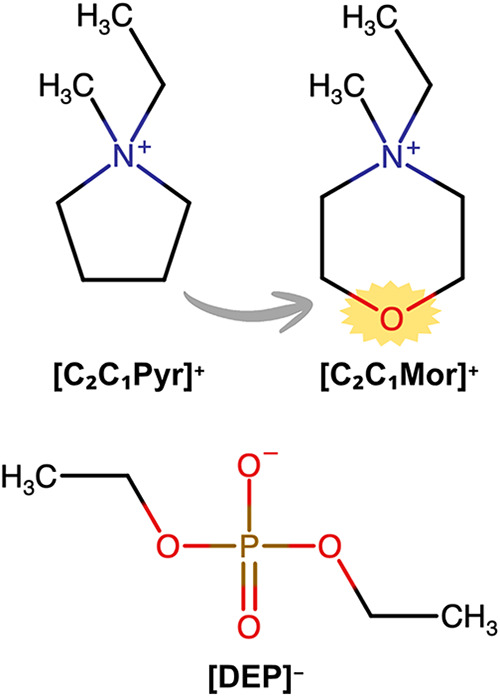
Chemical structures of the ions forming the investigated ionic liquids: 1-ethyl-1-methylpyrrolidinium ([C_2_C_1_Pyr]^+^), 4-ethyl-4-methylmorpholinium ([C_2_C_1_Mor]^+^), and diethyl phosphate ([DEP]^−^). The highlighted oxygen atom indicates the structural modification distinguishing the morpholinium cation from its pyrrolidinium analogue.

## Experimental Procedures

### Materials

The ILs investigated in this work, 1-ethyl-1-methylpyrrolidinium diethyl phosphate and 4-ethyl-4-methylmorpholinium diethyl phosphate, hereafter abbreviated as [C_2_C_1_Pyr]­[DEP] and [C_2_C_1_Mor]­[DEP], were synthesized and purified according to procedures described in detail in our previous work.^33^

All molecular solutes were obtained from commercial suppliers (Sigma-Aldrich or Fluka) and used as received without further purification. Similar set of solutes, together with supplier information and declared purities, has been employed in our earlier IDAC studies,^12,32,34^ ensuring consistency and comparability of the experimental data set. The selected compounds span a broad range of chemical functionalities, polarities, and molecular sizes, including *n*-alkanes, cycloalkanes, alkenes, alkynes, aromatics, alcohols, ketones, ethers, nitriles, nitro compounds, and heterocycles.

### Determination of IDACs

IDACs were determined from gas–liquid chromatography (GLC) retention data using the classical Everett-Cruickshank methodology.^35,36^ Full derivations of the working equations, as well as detailed descriptions of the experimental setup and measurement protocol, have been reported previously^12,32,34^ and are not repeated here.

Packed GC columns were prepared by coating an inert solid support with the studied IL dissolved in a volatile solvent, followed by controlled solvent evaporation. Two columns with different IL mass loadings were used for each system: 45.0% (8.854 mmol) and 50.6% (9.836 mmol) for [C_2_C_1_Pyr]­[DEP], and 40.2% (5.937 mmol) and 45.3% (7.214 mmol) for [C_2_C_1_Mor]­[DEP]. The lower loadings employed for [C_2_C_1_Mor]­[DEP] reflect its higher viscosity, which restricts uniform film formation at elevated IL contents. The selected loading ranges ensured uniform coating of the support; higher IL contents led to nonuniform films due to the high viscosity and surface tension of the ILs, resulting in localized accumulation and partial pore blocking, which adversely affected retention reproducibility.

Prior to measurements, all columns were conditioned at elevated temperature until constant mass was achieved to remove residual volatile impurities. No measurable mass loss was observed during subsequent experiments within the accuracy of the analytical balance (±0.0005 g). Retention measurements for each solute at a given temperature were performed repeatedly using different columns, and the reported IDAC values represent averages over independent determinations. No systematic dependence of the measured retention factors on IL loading was observed within the investigated range, confirming the absence of significant mass-transfer limitations.

Repeatability analysis indicated moderate variability between columns, with mean absolute percentage errors typically at or below 5% for most systems. Larger deviations were observed primarily for weakly retained hydrocarbons and selected alkenes at lower temperatures, whereas strongly interacting polar solutes exhibited substantially better reproducibility, often below 1%. Based on these results and comparison with literature data obtained using analogous GLC methodologies, the overall relative uncertainty of the reported IDAC values is estimated to be within (5 to 10)%.^37^

## Modeling

This section summarizes the modeling approaches used to correlate, predict, and interpret the experimentally determined IDACs in the investigated ILs. Unless stated otherwise, subscripts 1 and 2 denote quantities referring to the IL and the molecular solute (either in the pure state or at infinite dilution in the IL), respectively. In particular, γ_2_^∞^ denotes the IDAC of the solute in the IL, *T* is the absolute temperature, and *R* = 8.314 J·K^–1^ is the universal gas constant.

### Linear Solvation-Energy Relationship

The linear solvation-energy relationship (LSER) methodology provides a quantitative framework for correlating thermodynamic properties associated with solute transfer between phases with molecular interaction parameters.^38^ In the present work, LSER analysis was applied to rationalize gas–liquid partitioning behavior of molecular solutes in both studied ILs on the basis of experimentally determined IDACs.^39,40^

Gas–liquid partition coefficients (*K*_L,2_) were calculated from γ_2_^∞^ values according to

1 $$K_{L,2}=\frac{\mathrm{RT}}{\gamma_{2}^{\infty }p_{2}^{0}V_{1}}$$

where *p*_2_^0^ denotes the saturated vapor pressure of the solute and *V*_1_ is the molar volume of the IL.^41^ The vapor pressures were calculated using the DIPPR 801 equations.^42^ The values of *V*_1_ = *M*_1_/ρ_1_ were determined from the following correlations obtained by regression of IL density data (ρ_1_) reported previously:^33^ ρ_1_/kg·m^–3^ = 1297.6 – 0.6364 *T*/K for [C_2_C_1_Pyr]­[DEP] (with *M*_1_ = 267.305 g·mol^–1^) and ρ_1_/kg·m^–3^ = 1351.1 – 0.6448 *T*/K for [C_2_C_1_Mor]­[DEP] (with *M*_1_ = 283.305 g·mol^–1^).

In the classical LSER representation, the decimal logarithm of the partition coefficient is expressed as

2 $$\log K_{L,2}=c_{1}+e_{1}E_{2}+s_{1}S_{2}+a_{1}A_{2}+b_{1}B_{2}+l_{1}L_{2}$$

where **E**_2_ is the excess molar refraction descriptor associated with interactions involving π and nonbonding electrons, **S**_2_ quantifies solute dipolarity/polarizability, **A**_2_ and **B**_2_ represent hydrogen-bond acidity and basicity of the solute, respectively, and **L**_2_ reflects dispersive interactions and cavity formation.^38^ The coefficients *c*_1_, *e*_1_, *s*_1_, *a*_1_, *b*_1_, and *l*_1_ are IL-specific parameters describing complementary interaction capabilities of a given IL toward the corresponding solute properties. Descriptor values were taken from the UFZ LSER database,^43^ and the coefficients were obtained by ordinary least-squares regression of experimental log *K*_L,2_ data at fixed temperature. The corresponding design matrix and regression results are provided in the Supporting Information (Sheets S2 and S3, respectively).

To account explicitly for temperature effects, a temperature-dependent LSER (TD-LSER) formulation^44,45^ was also employed, in which IL-specific coefficients are expressed as linear functions of reciprocal temperature

3 $$\log K_{L,2}={c_{1}}^{\prime }+\frac{c_{1}^{\prime \prime }}{T/K}+\left({e_{1}}^{\prime }+\frac{e_{1}^{\prime \prime }}{T/K}\right)E_{2}+\left({s_{1}}^{\prime }+\frac{s_{1}^{\prime \prime }}{T/K}\right)S_{2}+\left({a_{1}}^{\prime }+\frac{a_{1}^{\prime \prime }}{T/K}\right)A_{2}+\left({b_{1}}^{\prime }+\frac{b_{1}^{\prime \prime }}{T/K}\right)B_{2}+\left({l_{1}}^{\prime }+\frac{l_{1}^{\prime \prime }}{T/K}\right)L_{2}$$

This formulation enables simultaneous correlation of data over a range of temperatures and provides insight into enthalpic contributions to solute–IL interactions. In the present study, the final descriptor set was selected using recursive feature elimination combined with cross-validation (RFECV), ensuring robust statistical performance while avoiding overfitting. The design matrix and statistical results for the TD-LSER analysis are provided in the Supporting Information (Sheets S4 and S5, respectively).

### Regular Solution Theory

The Hildebrand solubility parameter for a compound (δ_H_) is a thermodynamic quantity widely used to assess cohesive interactions and mutual affinity between fluids. Within the framework of the Scatchard–Hildebrand regular solution theory (RST), it is defined as

4 $$\delta_{H}\equiv {\left(\frac{\Delta_{l}^{v}H-\mathrm{RT}}{V}\right)}^{1/2}$$

where Δ_l_^v^ *H* is the molar enthalpy of vaporization and *V* is the molar volume of the fluid. The parameter δ_H_ reflects the cohesive energy density and thus the overall strength of intermolecular interactions.

For molecular solutes, the required thermophysical properties were estimated using standard DIPPR 801 correlations.^42^ For ILs, direct evaluation of δ_H,1_ from eq 4 is generally not feasible due to their negligible volatility. Instead, δ_H,1_ was estimated from γ_2_^∞^ data by combining the Scatchard–Hildebrand energetic term with the Flory combinatorial contribution accounting for size asymmetry, following procedures established in our previous work.^12,32,34^

The combinatorial contribution to the IDAC is given by

5 $$\ln\gamma_{2}^{\infty ,\mathrm{comb}}=1-\frac{V_{2}}{V_{1}}+\ln\frac{V_{2}}{V_{1}}$$

and the Flory–Huggins interaction parameter

6 $$\chi_{12}=\frac{V_{1}}{\mathrm{RT}}{\left(\delta_{H,1}-\delta_{H,2}\right)}^{2}$$

is obtained as the residual contribution to the IDAC (γ_2_^∞,res^), calculated as

7 $$\chi_{12}=\ln\gamma_{2}^{\infty ,\mathrm{res}}=\ln\gamma_{2}^{\infty }-\ln\gamma_{2}^{\infty ,\mathrm{comb}}$$

Rearrangement of (eqs 6 and 7) yields the following expression for the quantity *Y*, which is linear in δ_H,2_ and allows estimation of δ_H,1_ from the slope and intercept

8 $$Y\equiv \frac{\delta_{H,2}^{2}}{\mathrm{RT}}-\frac{\chi_{12}}{V_{2}}=\frac{2\delta_{H,1}}{\mathrm{RT}}\delta_{H,2}-\frac{\delta_{H,1}^{2}}{\mathrm{RT}}$$

Linear regression of *Y* against δ_H,2_ yields δ_H,1_ independently from both the slope and intercept; agreement between these estimates provides a measure of internal consistency of the γ_2_^∞^ data set. The underlying data used for the determination of δ_H,1_ are compiled in the Supporting Information (Sheet S6). For construction of the *Y* vs δ_H,2_ relationships at selected temperatures, IDAC values not directly available from experiment were obtained by linear extrapolation of ln γ_2_^∞^ versus 1/*T* relationships established from measured data, ensuring thermodynamic consistency of the data set.

### Conductor-like Screening Model for Real Solvents

The conductor-like screening model for real solvents (COSMO-RS) represents a hybrid methodology combining quantum chemistry with statistical thermodynamics, widely used to predict and analyze phase behavior and solvation energetics across diverse molecular and ionic systems.^46–48^ In recent years, the model has undergone extensive validation for IL-based systems.^49–55^ Within this framework, molecules are represented as collections of surface segments characterized by their screening charge density (σ), obtained from quantum-chemical calculations using the COSMO approach.^56^ The resulting σ-profile, *p*(σ), constitutes the primary molecular descriptor and enables calculation of segment-based interaction energies and macroscopic thermodynamic properties.

In the present work, COSMO-RS was used both for direct prediction of IDAC values and for interpretation of experimental trends through physically meaningful descriptors derived from σ-profiles. This approach enables direct linkage between macroscopic thermodynamic behavior and microscopic surface polarity distributions.

The σ-profiles were first normalized into a dimensionless distribution

9 $$\mathrm{p̃}\left(\sigma \right)\equiv \frac{p\left(\sigma \right)}{\int p\left(\sigma \right)d\sigma }$$

which satisfies ∫*p̃*_2_(σ)­dσ = 1. Based on this representation, the following descriptors were defined

10a $${\mathrm{\sigma ̅}}_{2}\equiv \int \sigma {\mathrm{p̃}}_{2}\left(\sigma \right)d\sigma$$

10b $$w_{\sigma ,2}\equiv [{\int (\sigma -{\mathrm{\sigma ̅}}_{2}{)}^{2}{\mathrm{p̃}}_{2}\left(\sigma \right)d\sigma ]}^{1/2}$$

10c $$A_{\mathrm{HBD},2}\equiv \int_{\sigma <-\sigma_{0}}{\mathrm{p̃}}_{2}\left(\sigma \right)d\sigma$$

10d $$A_{\mathrm{HBA},2}\equiv \int_{\sigma >+\sigma_{0}}{\mathrm{p̃}}_{2}\left(\sigma \right)d\sigma$$

10e $$O_{2}^{\pm }\equiv \int {\mathrm{p̃}}_{2}\left(\sigma \right){\mathrm{p̃}}_{1}^{\pm }\left(-\sigma \right)d\sigma$$

10f $$\Pi_{2}\equiv \frac{O_{2}^{-}-O_{2}^{+}}{O_{2}^{-}+O_{2}^{+}}$$

with the threshold set at σ_0_ = 0.8 *e*·nm^–2^. The average surface charge density σ̅_2_ characterizes net polarity, while *w*_σ,2_ quantifies polarity heterogeneity. The terms *A*_HBD,2_ and *A*_HBA,2_ represent hydrogen-bond donor and acceptor surface fractions, respectively. The overlap terms *O*_2_^±^ evaluate electrostatic complementarity between the solute and the ionic species of the IL, and the preference index Π_2_ indicates whether solvation is dominated by interactions with the cation or the anion.

To account for dispersive and steric effects, the COSMO cavity surface area (*A*_COSMO,2_) and volume (*V*_COSMO,2_) were used as molecular size descriptors.

All COSMO-RS calculations were performed using the COSMO*therm* package.^57^ COSMO input files were generated at different levels of theory (BP-SVP-AM1-COSMO, BP-TZVP-COSMO, and BP-TZVPD-FINE)^58–61^ using the BP exchange-correlation functional within TURBOMOLE (version 7.2).^62^ In these designations, BP denotes the Becke-Perdew functional, SVP and TZVP/TZVPD refer to split-valence and triple-ζ basis sets, respectively, AM1-COSMO indicates semiempirical geometry optimization, and FINE corresponds to a refined surface grid. The use of multiple parametrization levels accounts for the sensitivity of COSMO-RS predictions to quantum-chemical settings. The corresponding σ-profile data are provided in the Supporting Information (Sheet S7A–C).

Conformer ensembles for both solutes and IL ions were generated using COSMO*conf* (version 4.2). The corresponding σ-profiles were averaged arithmetically over the generated conformers and used for descriptor evaluation. Descriptor integrals were evaluated numerically over the range of σ from −3 to 3 *e*·nm^–2^ using the trapezoidal rule. This procedure provided a robust set of descriptors for correlation analysis of the IDAC data and a physically consistent framework for interpreting solute–IL interactions in terms of polarity, hydrogen bonding, and electrostatic complementarity.

## Results and Discussion

### Experimental Data

The complete temperature-dependent data sets are reported in Tables 1 and 2; the corresponding gas–liquid partition coefficients calculated from these data are provided in the Supporting Information (Sheet S1). This section analyzes the experimentally determined IDACs for molecular solutes in the two investigated ILs. The discussion is organized into three parts. First, solute structure effects at a representative temperature (*T* = 338.15 K) are examined to identify systematic trends across homologous series and functional groups. Next, the temperature dependence of γ_2_^∞^ is analyzed, and the corresponding excess thermodynamic functions derived from these data are discussed. Finally, the impact of cation functionalization is assessed by direct comparison of the two ILs, integrating insights from both structural and temperature-dependent analyses.

**Table 1:** Experimental Infinite Dilution Activity Coefficients (*γ*_2_^∞^) of Molecular Solutes in 1-Ethyl-1-Methylpyrrolidinium Diethylphosphate IL as a Function of Temperature (*T*)

	γ_2_^∞^(*T*)					
solute	308.15 K	318.15 K	328.15 K	338.15 K	348.15 K	358.15 K
*n*-heptane	40.8	38.0	35.5	33.6	31.8	30.0
*n*-octane	53.1	49.7	46.8	44.0	41.8	39.7
*n*-nonane	71.6	67.0	62.8	59	55.5	52.8
*n*-decane	101	92.8	86.0	80.7	75.5	70.5
cycloheptane	17.9	16.9	16.0	15.2	14.6	13.9
cyclooctane	22.4	20.9	19.8	18.7	17.8	17.0
1-hexene	16.9	16.0	15.2	14.6	14.1	13.5
1-heptene	21.6	20.8	19.9	19.2	18.5	18.0
1-octene	29.6	28.2	27.1	25.9	24.8	23.9
1-decene	53.8	51.1	48.8	46.6	44.7	42.9
cyclohexene	8.46	8.14	7.83	7.60	7.36	7.14
1-pentyne	1.41	1.51	1.61	1.73	1.84	1.94
1-hexyne	1.91	2.05	2.2	2.35	2.50	2.62
1-heptyne	2.60	2.79	2.96	3.17	3.34	3.53
1-octyne	3.6	3.82	4.05	4.29	4.51	4.73
benzene	2.06	2.06	2.08	2.08	2.10	2.10
toluene	3.42	3.42	3.44	3.45	3.45	3.46
ethylbenzene	5.09	5.05	5.03	5.00	4.96	4.93
*o*-xylene	5.11	5.05	5.00	4.95	4.91	4.88
*m*-xylene	5.96	5.87	5.78	5.70	5.62	5.55
*p*-xylene	5.79	5.73	5.68	5.62	5.58	5.54
methanol				0.0290	0.0320	0.0355
ethanol				0.0505	0.0550	0.0600
acetone	1.77	1.75	1.75	1.75	1.73	1.73
2-pentanone	3.00	3.01	3.03	3.04	3.04	3.05
3-pentanone	3.04	3.05	3.06	3.08	3.08	3.10
2-hexanone	4.06	4.03	4.00	3.98	3.95	3.93
3-hexanone	4.49	4.45	4.42	4.38	4.33	4.30
thiophene	0.926	0.966	0.999	1.04	1.07	1.11
tetrahydrofuran	2.80	2.75	2.69	2.65	2.60	2.56
1,4-dioxane	2.08	2.07	2.07	2.06	2.06	2.06
acetonitrile	0.641	0.656	0.671	0.684	0.699	0.710
pyridine	0.967	0.985	0.995	1.01	1.03	1.04
1-nitropropane	1.00	1.03	1.06	1.09	1.11	1.14

**Table 2:** Experimental Infinite Dilution Activity Coefficients (*γ*_2_^∞^) of Molecular Solutes in 4-Ethyl-4-Methylmorpholinium Diethylphosphate IL as a Function of Temperature (*T*)

	γ_2_^∞^(*T*)					
solute	308.15 K	318.15 K	328.15 K	338.15 K	348.15 K	358.15 K
*n*-heptane	116	92.8	75.5	62.7	53	45.2
*n*-octane	126	106	88.9	76.0	66.2	57.4
*n*-nonane	148	128	109	96.2	85.8	76.9
*n*-decane	180	160	140	126	114	103
cycloheptane	36.6	31.4	27.3	23.8	21.4	19.2
cyclooctane	39.7	35.0	30.8	27.5	24.9	22.8
1-hexene	44.7	36.9	30.9	26.0	22.4	19.4
1-heptene	51.9	44.1	37.9	32.8	28.9	25.4
1-octene	60.6	53.4	48.0	43.0	38.8	35.6
1-decene	91.9	84.0	76.8	71.2	66.4	61.8
cyclohexene	16.2	14.3	12.8	11.5	10.4	9.57
1-pentyne	3.08	3.08	3.06	3.05	3.04	3.04
1-hexyne	4.13	4.12	4.12	4.12	4.11	4.11
1-heptyne	5.29	5.34	5.38	5.44	5.49	5.54
1-octyne	7.14	7.25	7.33	7.41	7.49	7.56
benzene	4.22	3.86	3.57	3.30	3.06	2.88
toluene	6.67	6.12	5.67	5.29	4.96	4.67
ethylbenzene	9.63	8.89	8.27	7.74	7.29	6.85
*o*-xylene	9.10	8.50	7.91	7.39	6.99	6.59
*m*-xylene	10.9	10.1	9.37	8.75	8.23	7.77
*p*-xylene	10.4	9.71	9.11	8.54	8.09	7.69
methanol			0.0445	0.0475	0.0515	0.0555
ethanol			0.0805	0.0865	0.0925	0.0985
acetone	2.58	2.43	2.28	2.16	2.04	1.94
2-pentanone	4.89	4.62	4.38	4.14	3.96	3.78
3-pentanone	4.97	4.72	4.49	4.28	4.09	3.93
2-hexanone	6.25	5.94	5.67	5.42	5.21	5.01
3-hexanone	7.55	7.04	6.62	6.22	5.89	5.61
thiophene	1.77	1.73	1.69	1.65	1.62	1.60
tetrahydrofuran	4.64	4.13	3.73	3.38	3.11	2.83
1,4-dioxane	2.83	2.70	2.56	2.46	2.35	2.27
acetonitrile	0.776	0.782	0.79	0.796	0.801	0.807
pyridine	1.44	1.40	1.38	1.33	1.31	1.28
1-nitropropane	1.60	1.58	1.58	1.58	1.56	1.56

#### Solute Structure Effects

At *T* = 338.15 K, both tabulated data and Figure 2 reveal clear and systematic trends with solute structure. For nonpolar hydrocarbons (Figure 2a–c), γ_2_^∞^ increases strongly with molecular size within each homologous series. For example, in the pyrrolidinium IL, γ_2_^∞^ increases from 33.6 for *n*-heptane to 80.7 for *n*-decane, whereas in the morpholinium IL the corresponding values rise from 62.7 to 126.0, corresponding to an increase of roughly from 1.6 to 1.9 times relative to the pyrrolidinium system, with the relative difference decreasing slightly with increasing chain length (Figure 2a). These trends are nearly linear in ln γ_2_^∞^ versus carbon number, indicating that solvation is dominated by the free-energy cost of cavity formation, which scales with solute size and reflects the high cohesive energy density of the IL. Similar behavior is observed for cycloalkanes (Figure 2b), confirming that dispersive interactions alone are insufficient to compensate for the energetic penalty associated with disrupting the strongly interacting ionic network.

**Figure 2:**
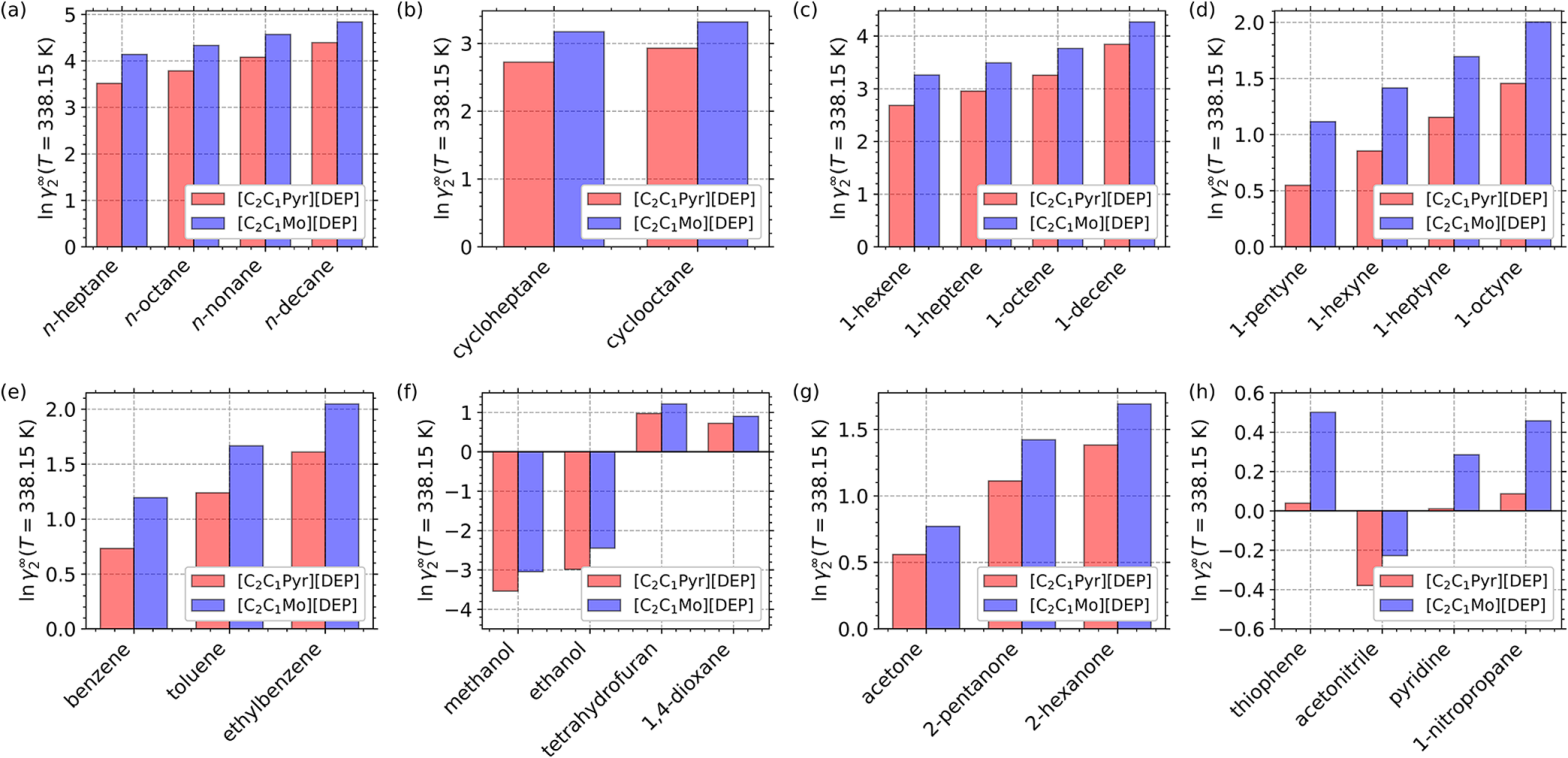
Infinite dilution activity coefficients (γ_2_^∞^) of molecular solutes in [C_2_C_1_Pyr]­[DEP] and [C_2_C_1_Mor]­[DEP] at *T* = 338.15 K as a function of solute structure: (a) *n*-alkanes; (b) cycloalkanes; (c) 1-alkenes; (d) 1-alkynes; (e) aromatic hydrocarbons; (f) alcohols and ethers; (g) ketones; (h) selected polar solutes.

At comparable carbon numbers, 1-alkenes (Figure 2c) exhibit significantly lower γ_2_^∞^ values than the corresponding *n*-alkanes, indicating additional stabilization due to π-electron interactions with the IL. 1-Alkynes (Figure 2d) show even lower values, consistent with enhanced interactions arising from their high polarizability and π-electron density; however, a clear increase with chain length remains, indicating that cavity formation still contributes alongside these specific interactions.

Aromatic hydrocarbons (Figure 2e) display intermediate behavior, with γ_2_^∞^ increasing systematically with alkyl substitution, reflecting the growing contribution of nonpolar surface area that counteracts favorable π-interactions.

Strongly polar solutes (Figure 2f–h) exhibit much lower γ_2_^∞^ values overall. Alcohols show extremely low values (on the order of 10^–2^), indicative of strong hydrogen-bonding interactions with the IL. In contrast, ethers such as tetrahydrofuran and 1,4-dioxane exhibit only moderately reduced values, consistent with weaker, predominantly hydrogen-bond-acceptor interactions, while ketones occupy an intermediate regime due to competition between polar carbonyl interactions and nonpolar alkyl groups. Nitriles, nitro compounds, and heterocycles are stabilized primarily through dipolar interactions. Several of these solutes (e.g., thiophene, pyridine) exhibit γ_2_^∞^ values close to unity, indicating nearly ideal solvation conditions where solute–solvent interactions closely match solvent–solvent interactions.

Overall, these trends demonstrate a transition from cohesion-dominated solvation for nonpolar solutes to interaction-driven solvation for polar compounds. Importantly, both ILs exhibit essentially identical qualitative trends, demonstrating that cation functionalization alters interaction magnitudes but does not change the underlying hierarchy of solvation mechanisms.

#### Temperature Dependence

The temperature dependence of γ_2_^∞^, shown in Figure 3, provides further insight into solvation mechanisms. The excess partial thermodynamic functions obtained from the temperature dependence of IDACs were evaluated from linear regression of the van’t Hoff-type relation

11 $$\ln\gamma_{2}^{\infty }=a_{2}+\frac{b_{2}}{T}$$

which assumes temperature-independent excess enthalpy and entropy over the investigated range. Under this assumption, the thermodynamic functions follow as *H̅*_2_^E,∞^ = *b*_2_ *R* and *S̅*_2_^E,∞^ = −*a*_2_ *R*, respectively.

**Figure 3:**
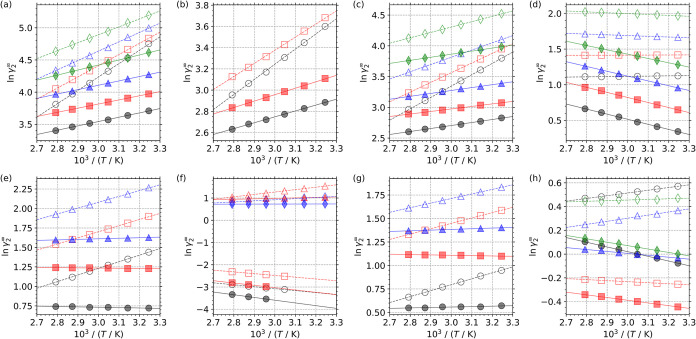
Temperature dependence of infinite dilution activity coefficients (ln γ_2_^∞^ vs 1/*T*) of molecular solutes in [C_2_C_1_Pyr]­[DEP] (filled symbols) and [C_2_C_1_Mor]­[DEP] (open symbols): (a) *n*-alkanes: *n*-heptane (circles), *n*-octane (squares), *n*-nonane (triangles), *n*-decane (diamonds); (b) cycloalkanes: cycloheptane (circles), cyclooctane (squares); (c) 1-alkenes: 1-hexene (circles), 1-heptene (squares), 1-octene (triangles), 1-decene (diamonds); (d) 1-alkynes: 1-pentyne (circles), 1-hexyne (squares), 1-heptyne (triangles), 1-octyne (diamonds); (e) aromatic hydrocarbons: benzene (circles), toluene (squares), ethylbenzene (triangles); (f) alcohols and ethers: methanol (circles), ethanol (squares), tetrahydrofuran (triangles), 1,4-dioxane (diamonds); (g) ketones: acetone (circles), 2-pentanone (squares), 2-hexanone (triangles); (h) selected polar solutes: thiophene (circles), acetonitrile (squares), pyridine (triangles), 1-nitropropane (diamonds).

The high linearity of ln γ_2_^∞^ versus 1/*T*, with *R* ^2^ values typically exceeding 0.99 (see Table 3), confirms that the representation by eq 16 is appropriate over the investigated temperature range. Nevertheless, for several systems—most notably aromatics, ketones, and ethers in the pyrrolidinium IL—more pronounced deviations from ideal linearity are observed (*R* ^2^ < 0.95), indicating that the assumption of temperature-independent enthalpic contributions is not strictly fulfilled. These deviations likely reflect temperature-dependent solute–IL interactions and subtle changes in liquid structure. In contrast, the morpholinium IL generally exhibits more consistent linear behavior, suggesting a more uniform solvation environment upon cation functionalization.

**Table 3:** Excess Partial Thermodynamic Functions at Infinite Dilution (*H̅*_**2**_^**E**,∞^, *S̅*_**2**_^**E**,∞^) and Coefficients of Determination (*R* ^2^) for Molecular Solutes in the Studied ILs Obtained from the Temperature Dependence of Infinite Dilution Activity Coefficients^a^

	[C_2_C_1_Pyr][DEP]			[C_2_C_1_Mor][DEP]		
solute	*H̅*_2_^E,∞^/kJ·mol^–1^	*S̅*_2_^E,∞^/J ·mol^–1^·K^–1^		*H̅*_2_^E,∞^ /kJ·mol^–1^	*S̅*_2_^E,∞^ /J·mol^–1^·K^–1^	*R* ^2^
*n*-heptane	5.58	–12.7	0.999	17.3	16.6	1.000
*n*-octane	5.34	–15.7	1.000	14.4	6.65	1.000
*n*-nonane	5.64	–17.2	1.000	12.1	–2.30	0.999
*n*-decane	6.51	–17.2	0.999	10.3	–9.84	0.999
cycloheptane	4.61	–9.02	0.999	11.9	8.61	0.999
cyclooctane	5.04	–9.47	0.999	10.3	2.74	0.999
1-hexene	4.05	–10.3	0.998	15.3	18.2	1.000
1-heptene	3.41	–14.5	0.999	13.1	9.65	1.000
1-octene	3.93	–15.4	0.999	9.79	–2.34	1.000
1-decene	4.14	–19.7	1.000	7.26	–14.0	1.000
cyclohexene	3.10	–7.69	1.000	9.70	8.37	1.000
1-pentyne	–5.93	–22.1	0.999	0.284	–8.44	0.931
1-hexyne	–5.89	–24.5	0.999	0.0829	–11.5	0.856
1-heptyne	–5.61	–26.1	0.999	–0.851	–16.6	0.995
1-octyne	–5.04	–27.0	1.000	–1.04	–19.7	0.998
benzene	–0.403	–7.30	0.912	7.05	10.9	1.000
toluene	–0.229	–11.0	0.923	6.52	5.42	1.000
ethylbenzene	0.575	–11.7	0.989	6.21	1.34	1.000
*o*-xylene	0.854	–10.8	0.996	5.95	0.947	1.000
*m*-xylene	1.31	–10.6	1.000	6.24	0.389	1.000
*p*-xylene	0.817	–11.9	0.998	5.57	–1.39	1.000
methanol	–10.2	–0.643	0.999	–7.26	3.81	0.996
ethanol	–8.67	–0.821	1.000	–6.57	0.915	1.000
acetone	0.391	–3.46	0.856	5.26	9.17	1.000
2-pentanone	–0.306	–10.1	0.944	4.74	2.18	1.000
3-pentanone	–0.350	–10.4	0.964	4.33	0.728	1.000
2-hexanone	0.598	–9.71	0.998	4.05	–2.07	1.000
3-hexanone	0.804	–9.89	0.991	5.47	0.954	1.000
thiophene	–3.29	–10.0	0.999	1.91	1.45	0.996
tetrahydrofuran	1.66	–3.19	0.999	8.98	16.4	1.000
1,4-dioxane	0.180	–5.49	0.863	4.09	4.63	0.999
acetonitrile	–1.89	–2.45	0.999	–0.723	–0.237	0.998
pyridine	–1.34	–4.08	0.991	2.16	3.98	0.989
1-nitropropane	–2.38	–7.74	0.998	0.433	–2.48	0.856

a Estimated from linear regression of ln γ_2_^∞^ versus 1/*T* according to eq 16 using data from Tables 1 and 2.

For nonpolar hydrocarbons (Figure 3a and b), ln γ_2_^∞^ decreases with increasing temperature, corresponding to positive *H̅*_2_^E,∞^ and negative *S̅*_2_^E,∞^. In the pyrrolidinium IL, *H̅*_2_^E,∞^ values fall in the range of approximately 5 to 7 kJ·mol^–1^, accompanied by strongly negative *S̅*_2_^E,∞^, showing that solvation is both energetically and entropically unfavorable due to the cost of cavity formation and the ordering of the IL around the solute. In contrast, the morpholinium IL exhibits substantially larger positive enthalpies, up to approximately 17 kJ·mol^–1^, while the entropic contribution becomes less negative or even positive. This combination suggests that the morpholinium-based IL is energetically more resistant to cavity formation while exhibiting reduced structural ordering constraints, leading to a solvation environment that is thermodynamically less favorable but entropically less restrictive.

Alkenes (Figure 3c) show similar qualitative behavior but with somewhat reduced enthalpic penalties compared to alkanes, consistent with additional stabilizing interactions involving the π-electron system. Alkynes (Figure 3d), however, exhibit markedly different behavior. In the pyrrolidinium IL, *H̅*_2_^E,∞^ is negative, approximately (−6 to −5) kJ·mol^–1^, indicating that favorable specific interactions outweigh the cost of cavity formation. Upon transition to the morpholinium IL, the enthalpic contribution shifts toward zero or slightly positive values, corresponding to a significant reduction in interaction strength. This change is accompanied by more negative entropic contributions, indicating that the solvation process becomes increasingly governed by structural constraints rather than energetic stabilization.

Aromatic solutes (Figure 3e) exhibit small enthalpic contributions in the pyrrolidinium IL, ranging from slightly negative to mildly positive values, together with moderately negative entropies. This behavior reflects a balance between dispersive interactions and weak specific stabilization. In the morpholinium IL, *H̅*_2_^E,∞^ increases systematically to values of approximately (5 to 7) kJ·mol^–1^, leading to enhanced temperature sensitivity and indicating that favorable interactions present in the pyrrolidinium system are partially diminished.

Strongly polar solutes (Figure 3f–h), particularly alcohols (Figure 3f), are characterized by large negative *H̅*_2_^E,∞^ and near-zero or slightly positive *S̅*_2_^E,∞^, demonstrating that their solvation is dominated by strong, specific interactions such as hydrogen bonding, with only minor structural penalties. In contrast, nitriles, ketones, and heterocycles (Figure 3g and h) show intermediate behavior, reflecting a competition between dipolar interactions and cohesion-related effects. For many of these solutes, the transition from pyrrolidinium to morpholinium shifts *H̅*_2_^E,∞^ toward more positive values, indicating a reduction in net interaction strength.

Overall, the temperature-dependent behavior confirms that cation functionalization systematically increases the enthalpic penalty of solvation while reducing or reversing the entropic penalty. These changes reflect a liquid environment in which solvation becomes energetically less favorable but structurally less restrictive, rather than a fundamental change in the types of interactions governing solvation.

#### Cation Functionalization Impact

The effect of cation functionalization is manifested as a systematic increase in γ_2_^∞^ upon transition from pyrrolidinium to morpholinium. At *T* = 338.15 K, this appears as a systematic upward shift in ln γ_2_^∞^ across most solute classes, most pronounced for nonpolar compounds and progressively diminishing with increasing solute polarity. For linear alkanes, the increase corresponds to a factor of approximately 1.6 to 2.1, whereas for strongly polar solutes the differences are much smaller.

Because the anion is identical in both systems, these differences arise from modifications of the cation structure. The insertion of an oxygen atom into the saturated ring increases cation polarity and introduces an additional localized dipole, which strengthens electrostatic cation–anion interactions and modifies the hydrogen-bonding environment. At the same time, the transition from a five-membered to a six-membered ring affects conformational flexibility and packing efficiency. Together, these factors increase the thermodynamic cost of accommodating nonpolar solutes and shift the balance of intermolecular interactions in the liquid toward stronger cohesive contributions.

The temperature-dependent analysis (Figure 3 and Table 3) supports this interpretation. In the morpholinium IL, nonpolar solutes exhibit substantially more positive excess enthalpies, indicating a greater energetic cost of disrupting the liquid structure. This effect is accompanied by less negative or even positive excess entropies, indicating that the entropic penalty associated with local structuring around the solute is reduced relative to the pyrrolidinium system. For unsaturated and aromatic solutes, similar but less pronounced trends are observed. In the case of polar solutes, the magnitude of favorable enthalpic contributions generally decreases, while entropic contributions become more positive or less negative, indicating a shift toward a greater relative importance of entropy in stabilizing these systems.

These observations demonstrate that cation functionalization primarily modifies the baseline thermodynamic cost of solvation through redistribution of intermolecular interactions in the liquid, rather than through a change in solvation mechanism or a strong enhancement of specific interactions. Consistently, the relative ordering of solute affinities is preserved between the two ILs, confirming that cation functionalization affects the magnitude rather than the nature of these interactions.

Comparison with literature data^19,21,23,27,29,31^ for related ILs supports the generality of these trends. Systems based on pyrrolidinium and morpholinium cations bearing a 2-methoxyethyl substituent with common [NTf_2_] ^19,27^ and [FAP] ^21,31^ anions exhibit analogous increases in γ_2_^∞^ for nonpolar solutes upon cation modification, while the effect diminishes for strongly polar compounds. A similar but more pronounced behavior is observed for [TCM]-based systems. In particular, comparison of 1-butyl-1-methylpyrrolidinium and 4-butyl-4-methylmorpholinium ILs reveals a substantial increase in γ_2_^∞^ for aliphatic and alicyclic hydrocarbons, as well as other weakly interacting solutes, upon insertion of an oxygen atom into the cation ring, whereas the corresponding changes for polar solutes are markedly smaller and less systematic.^23,29^ These observations consistently demonstrate that modification of the cation core provides a systematic means of tuning solute–IL interactions.

Overall, the results highlight two primary factors governing solvation behavior in ILs: the liquid-state interaction balance and the strength of specific solute–IL interactions. Nonpolar solutes primarily probe the energetic cost of disrupting the IL environment, whereas polar solutes reflect the balance of specific interactions. Cation functionalization modulates both contributions in a predictable and chemically interpretable manner, primarily by increasing the thermodynamic cost of solvation while shifting the enthalpy–entropy balance.

### Modeling

This section analyzes the experimental IDAC data using complementary modeling approaches of increasing levels of physical detail. First, LSER analysis is used to interpret gas–liquid partitioning in terms of dispersive, polarity/polarizability, and hydrogen-bonding contributions, both at a representative temperature and over the full investigated temperature range. Next, regular solution theory is applied to estimate effective Hildebrand solubility parameters from the IDAC data. Finally, COSMO-RS is used to rationalize the observed trends at the molecular level through σ-profile analysis, predictive performance evaluation, and descriptor-based correlations. Together, these approaches provide a consistent picture of how cation functionalization modifies solvation thermodynamics in the studied ILs.

#### LSER

##### Gas–Liquid Partition Coefficients

The gas–liquid partition coefficients, eq 1, exhibit a clear and systematic dependence on cation structure. Replacement of the pyrrolidinium core by morpholinium results in consistently lower *K*_L,2_ values for all solutes across the investigated temperature range. At *T* = 338.15 K, for example, *K*_L,2_ decreases from 71.95 to 45.52 for *n*-decane, from 147.00 to 94.69 for toluene, and from 281.94 to 239.29 for acetonitrile. The magnitude of this effect is therefore largest for nonpolar hydrocarbons, for which reductions of roughly (35 to 45) % are observed, and progressively smaller for more polar solutes, where the decrease is typically below 20%. This behavior indicates that insertion of an oxygen atom into the cation ring reduces the affinity of the IL toward neutral solutes in gas-to-liquid transfer, with the strongest effect observed for compounds interacting predominantly through dispersion forces. For polar solutes, the smaller changes in *K*_L,2_ suggest that the reduced nonspecific affinity is at least partially compensated by favorable dipolar or hydrogen-bonding interactions. To quantify these effects, the experimental partition coefficients were analyzed using the LSER approach, which enables systematic evaluation of cavity formation and dispersion contributions, polarity/polarizability effects, and specific interaction terms.

##### Correlation at *T* = 338.15 K

The final LSER correlations obtained for the studied ILs at *T* = 338.15 K—corresponding to the lowest temperature at which complete data sets were available for all investigated solutes—read as

12 $$\log K_{L,2}=-0.766\left(0.172\right)+0.141\left(0.146\right)E_{2}+2.125\left(0.196\right)S_{2}+6.680\left(0.351\right)A_{2}-0.202\left(0.242\right)B_{2}+0.551\left(0.045\right)L_{2}$$

for the [C_2_C_1_Pyr]­[DEP] IL, and

13 $$\log K_{L,2}=-1.019\left(0.178\right)+0.141\left(0.151\right)E_{2}+2.181\left(0.203\right)S_{2}+6.490\left(0.364\right)A_{2}-0.055\left(0.251\right)B_{2}+0.556\left(0.046\right)L_{2}$$

for the [C_2_C_1_Mor]­[DEP] IL. In both cases, the standard errors of the coefficients are given in parentheses, the number of solutes is *N* = 34, and the statistical parameters are *R* ^2^ = 0.960 (pyrrolidinium) and 0.959 (morpholinium), with adjusted *R* ^2^ = 0.953 and 0.952, and Fisher statistics *F* = 134 and 131, respectively. These values indicate a high level of statistical significance and confirm that the Abraham model provides a robust description of the gas–liquid partitioning behavior derived from the measured IDACs.

The statistical significance of the descriptors is consistent for both ILs. The coefficients associated with polarity/polarizability (*s*_1_), hydrogen-bond acidity (*a*_1_), and dispersion interactions (*l*_1_) are highly significant (*p* < 0.01), whereas the coefficients corresponding to excess molar refraction (*e*_1_) and hydrogen-bond basicity (*b*_1_) are statistically insignificant (*p* > 0.05). This indicates that solvation in the studied systems is dominated by nonspecific polar and dispersive interactions together with strong hydrogen-bond donor contributions of the IL, while the contribution of solute hydrogen-bond basicity is negligible. The retention of all descriptors in the final models reflects the use of a consistent descriptor set and enables direct comparison between the two ILs.

A direct comparison of the regression coefficients reveals that the two ILs exhibit remarkably similar interaction profiles. The values of the *s*_1_, *a*_1_, and *l*_1_ coefficients differ only within their respective uncertainties, demonstrating that the relative contributions of polarity/polarizability, hydrogen-bonding, and dispersion interactions remain essentially unchanged upon replacing the pyrrolidinium core with morpholinium. In both systems, the large positive *a*_1_ coefficient identifies hydrogen-bond donor interactions of the IL as a major stabilizing contribution, whereas the substantial *l*_1_ term reflects the importance of cavity formation and dispersion interactions. Together, these features are consistent with anion-dominated hydrogen-bond acceptance combined with a significant energetic penalty associated with accommodating the solute in the IL.

The primary effect of the structural modification is therefore reflected in the intercept term, which decreases from −0.766 to −1.019, corresponding to a nearly uniform reduction in log *K*_L,2_ values for all solutes. Importantly, the preservation of the relative magnitudes of the regression coefficients, together with the nearly identical ordering of *K*_L,2_ values across the solute set for both ILs, confirms that cation functionalization does not alter interaction selectivity. Instead, it produces a broadly similar downward shift in solvent affinity across chemically diverse solutes, reducing the overall solvent affinity through an increase in the baseline thermodynamic cost of solvation.

The quality of the LSER correlations is further illustrated in Figure 4. For both ILs, the data at *T* = 338.15 K (gray circles) are closely aligned along the line of perfect agreement, indicating that the model accurately captures the variation of *K*_L,2_ across different classes of solutes. The relatively small scatter observed over nearly 3 orders of magnitude confirms that the selected descriptors provide a consistent and physically meaningful representation of solute–IL interactions at this temperature.

**Figure 4:**
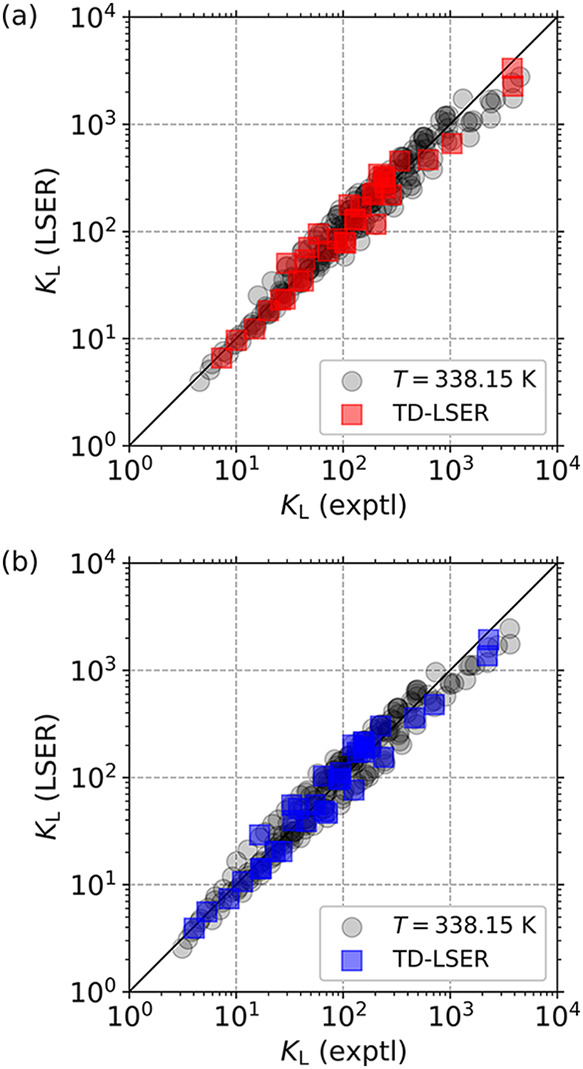
Parity plots comparing experimental and LSER-predicted gas–liquid partition coefficients (*K*_L,2_) over the investigated temperature range: (a) 1-ethyl-1-methylpyrrolidinium diethyl phosphate; (b) 4-ethyl-4-methylmorpholinium diethyl phosphate. The solid line represents perfect agreement.

##### Temperature-Dependent Correlation

To account explicitly for temperature effects, a temperature-dependent LSER (TD-LSER) formulation was employed according to eq 3. It should be noted that only the temperature-dependent contributions to the LSER coefficients are retained in the final correlations. The temperature-independent terms (*e*_1_^′^, *s*_1_^′^, *a*_1_^′^, *b*_1_^′^, and *l*_1_^′^) were systematically eliminated during model selection using the RFECV procedure, indicating that their inclusion does not improve the predictive performance of the model. This result indicates that the solvent response to the considered solute descriptors is dominated by enthalpic terms scaling with reciprocal temperature, whereas temperature-independent contributions are negligible over the investigated range.

The final correlations for the studied ILs are

14 $$\log K_{L,2}=-3.220\left(0.174\right)+\frac{826.1\left(61.1\right)}{T/K}+\frac{29.22\left(18.13\right)}{T/K}E_{2}+\frac{738.7\left(24.4\right)}{T/K}S_{2}+\frac{2181.3\left(50.5\right)}{T/K}A_{2}-\frac{98.92\left(30.28\right)}{T/K}B_{2}+\frac{189.5\left(5.5\right)}{T/K}L_{2}$$

for the [C_2_C_1_Pyr]­[DEP] IL, and

15 $$\log K_{L,2}=-2.578\left(0.186\right)+\frac{508.6\left(65.5\right)}{T/K}+\frac{28.85\left(19.38\right)}{T/K}E_{2}+\frac{764.3\left(26.0\right)}{T/K}S_{2}+\frac{2180.2\left(50.7\right)}{T/K}A_{2}-\frac{44.34\left(32.26\right)}{T/K}B_{2}+\frac{194.2\left(5.9\right)}{T/K}L_{2}$$

for the [C_2_C_1_Mor]­[DEP] IL. In both cases, the number of data points is *N* = 198 (pyrrolidinium) and 200 (morpholinium), and the statistical parameters are *R* ^2^ = 0.961 and 0.959, with adjusted *R* ^2^ = 0.960 and 0.958, and Fisher statistics *F* = 786 and 752, respectively. These values confirm the robustness of the TD-LSER description over the full investigated temperature range.

The parity plots in Figure 4 further demonstrate the predictive capability of the TD-LSER model. In Figure 4a (pyrrolidinium IL) and Figure 4b (morpholinium IL), the calculated values closely follow the line of perfect agreement over nearly 3 orders of magnitude in *K*_L,2_. The TD-LSER predictions at *T* = 338.15 K (colored squares) reproduce the experimental data with accuracy comparable to the temperature-specific LSER correlations, while simultaneously capturing the temperature dependence of the partition coefficients. The slightly increased scatter at higher *K*_L,2_ values is consistent with the larger absolute uncertainties associated with strongly nonideal systems.

The dominant temperature-dependent contributions arise from polarity/polarizability (*s*_1_^″^), hydrogen-bond acidity (*a*_1_^″^), and dispersion interactions (*l*_1_^″^), all of which are highly significant (*p* < 0.01). The excess molar refraction term (*e*_1_^″^) is statistically insignificant in both cases, while the hydrogen-bond basicity term (*b*_1_^″^) is significant only for the pyrrolidinium IL and becomes statistically insignificant for the morpholinium analogue.

Importantly, the temperature-dependent coefficients associated with *s*_1_^″^, *a*_1_^″^, and *l*_1_^″^ remain very similar for both ILs, demonstrating that the enthalpic contributions to solvation are governed by the same underlying interaction mechanisms. The primary difference is observed in the baseline 1/*T* term, which is significantly smaller for the [C_2_C_1_Mor]­[DEP] IL. This indicates a reduced overall enthalpic driving force for solvation, consistent with the lower partition coefficients observed experimentally.

These results confirm that cation functionalization does not qualitatively alter the nature or temperature dependence of solute–solvent interactions, but instead uniformly reduces their magnitude through a shift in the baseline thermodynamic driving force for solvation.

#### RST

The Hildebrand solubility parameters of the ILs, estimated from IDACs using the Scatchard–Hildebrand RST approach (see eq 8), are summarized in Table 4. For construction of the *Y* versus δ_H,2_ relationships at each temperature, missing γ_2_^∞^ values were obtained from linear extrapolation of van’t Hoff-type correlations of ln γ_2_^∞^ versus 1/*T*, ensuring internal thermodynamic consistency of the data set.

**Table 4:** Hildebrand Solubility Parameters (*δ*_H,1_) of ILs Estimated from Infinite Dilution Activity Coefficients as a Function of Temperature (*T*), together with Coefficients of Determination (*R* ^2^) of the Linear Regressions^a^

	δ_H,1_/MPa^1/2^			
*T*/K	slope	intercept	mean	*R* ^2^
[C_2_C_1_Pyr][DEP]				
308.15	28.0	26.8	27.4	0.958
318.15	27.7	26.5	27.1	0.958
328.15	27.3	26.2	26.8	0.957
338.15	27.0	25.9	26.4	0.957
348.15	26.6	25.5	26.1	0.956
358.15	26.2	25.2	25.7	0.955
[C_2_C_1_Mor][DEP]				
308.15	27.6	26.8	27.2	0.962
318.15	27.2	26.4	26.8	0.962
328.15	26.8	26.1	26.4	0.962
338.15	26.5	25.7	26.1	0.962
348.15	26.1	25.3	25.7	0.962
358.15	25.7	25.0	25.3	0.961

a Estimated from eq 8 (details in text).

For both systems, δ_H,1_ falls in the range of approximately (25 to 27) MPa^1/2^ and decreases systematically with increasing temperature. This trend reflects the expected reduction in cohesive energy density with increasing temperature. At all temperatures, the IL based on 1-ethyl-1-methylpyrrolidinium exhibits consistently higher δ_H,1_ values than its 4-ethyl-4-methylmorpholinium analogue. The differences between the two systems are on the order of (0.3 to 0.5) MPa^1/2^, indicating a small but systematic decrease in cohesive energy density upon insertion of an oxygen atom into the saturated cation ring.

At the same time, the magnitude of this difference is comparable to the discrepancy between δ_H,1_ values obtained independently from the slope and intercept of eq 8. This reflects the inherent uncertainty associated with the RST-based estimation procedure and indicates that the effect of cation functionalization, while consistent, is relatively subtle within the regular solution framework. The corresponding coefficients of determination, *R* ^2^ approximately from 0.95 to 0.96 for all cases, confirm good linearity of the *Y* versus δ_H,2_ relationships, with slightly higher *R* ^2^ values observed for the morpholinium-based system, suggesting behavior closer to that of an idealized regular solution.

The observed decrease in δ_H,1_ for the morpholinium IL can be reconciled with the IDAC trends only within the limitations of the regular solution model. The Hildebrand parameter represents an effective measure of cohesive energy density based on nonspecific interactions and does not explicitly account for directional or specific interactions such as hydrogen bonding or local electrostatic structuring. As a result, a decrease in δ_H,1_ does not necessarily imply improved solubility of nonpolar solutes. In the present systems, insertion of the ring oxygen slightly reduces the effective cohesive energy density but simultaneously alters the balance of intermolecular interactions, leading to less favorable incorporation of nonpolar solutes and consequently higher γ_2_^∞^ values.

Overall, the RST analysis indicates that cation functionalization produces only a modest change in the effective Hildebrand solubility parameter, while the experimentally observed changes in solvation behavior are significantly larger. This highlights the limited applicability of the regular solution framework to ILs, where solvation is governed by a complex interplay of dispersive, polar, and specific interactions that cannot be fully captured by a single scalar parameter. In particular, the small change in δ_H,1_ contrasts with the much larger differences in γ_2_^∞^ and excess enthalpies, indicating that the dominant effect of cation functionalization arises from redistribution of directional and electrostatic interactions rather than from changes in nonspecific cohesive energy density.

#### COSMO-RS

##### σ-Profile Analysis

The σ-profiles shown in Figure 5 provide a compact representation of the surface charge density distributions of the ions forming the studied ILs and, consequently, of their polarity and hydrogen-bonding characteristics. Negative σ values correspond to electron-deficient surface segments (hydrogen-bond donor regions), whereas positive σ values indicate electron-rich regions (hydrogen-bond acceptors). The central region around σ ≈ 0 reflects nonpolar surface contributions dominated by dispersion interactions.

**Figure 5:**
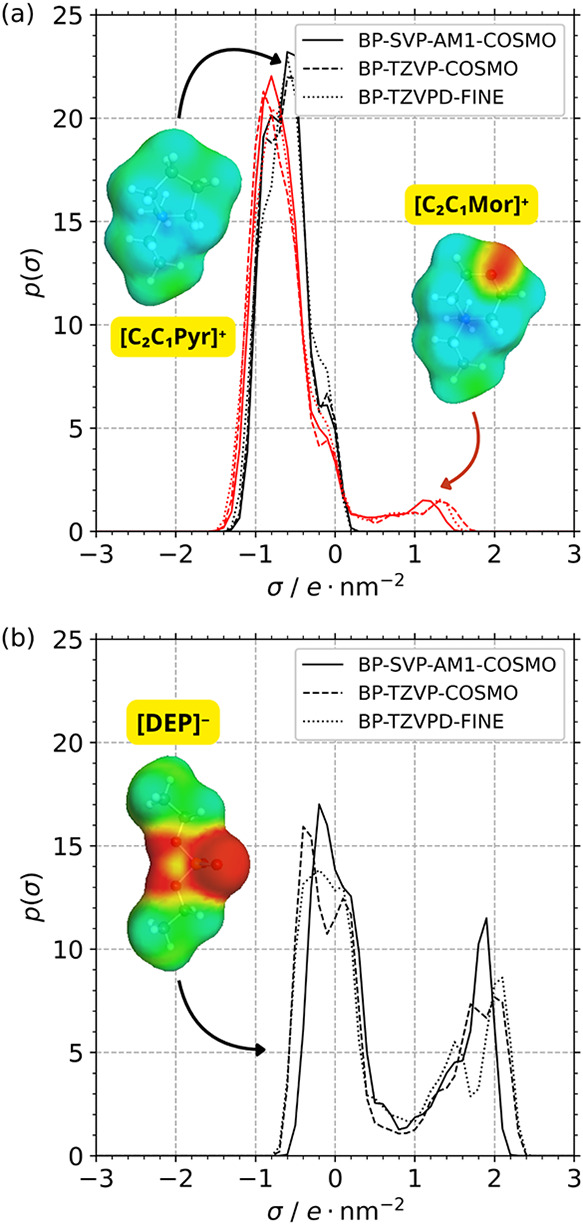
σ-profiles, *p*(σ), of the ions forming the studied ILs obtained from COSMO-RS calculations at different levels of theory: (a) comparison of the cations, 1-ethyl-1-methylpyrrolidinium [C_2_C_1_Pyr]^+^ (black lines) and 4-ethyl-4-methylmorpholinium [C_2_C_1_Mor]^+^ (red lines); (b) diethyl phosphate anion ([DEP]^−^). Solid, dashed, and dotted lines correspond to the BP-SVP-AM1-COSMO, BP-TZVP-COSMO, and BP-TZVPD-FINE levels of theory, respectively. Insets show the corresponding COSMO surface charge density distributions, where red denotes electron-rich regions (positive σ) and blue denotes electron-deficient regions (negative σ).

For the cations, both the 1-ethyl-1-methylpyrrolidinium and 4-ethyl-4-methylmorpholinium ions exhibit dominant contributions in the nonpolar region, consistent with their saturated alkyl substituents and overall weak polarity. However, clear differences emerge upon closer inspection. The pyrrolidinium cation shows a relatively narrow distribution centered near σ = 0, indicating a predominantly apolar surface with only limited polarity. In contrast, the morpholinium cation displays a broader σ-profile that is shifted toward positive σ values, with a noticeable extension into the hydrogen-bond acceptor region. This feature originates from the presence of the ether oxygen atom in the ring, which introduces localized electron density and enhances the hydrogen-bond acceptor character of the cation. These features indicate that insertion of the ring oxygen enhances localized electrostatic interactions within the ionic network, thereby increasing the thermodynamic cost of disrupting the liquid structure during solvation. This broadening is accompanied by a reduction in peak intensity around σ ≈ 0, indicating a more heterogeneous surface charge distribution, while changes in the negative σ region are comparatively minor.

The σ-profile of the diethyl phosphate anion is markedly different. It is strongly skewed toward positive σ values, reflecting the high electron density localized on the phosphoryl and alkoxy oxygen atoms. This indicates a dominant hydrogen-bond acceptor character, with only minor contributions in the negative σ region, highlighting its key role as a hydrogen-bond acceptor in solute–IL interactions. This interpretation is fully consistent with the LSER analysis, which identifies a strong contribution of the hydrogen-bond acidity descriptor (*a*_1_) and negligible contribution of solute hydrogen-bond basicity (*b*_1_).

Taken together, the σ-profiles show that replacing the pyrrolidinium core with morpholinium redistributes surface polarity by introducing localized polar sites while retaining substantial nonpolar surface area. This increased polarity heterogeneity is consistent with the LSER results, which show nearly unchanged interaction coefficients but a shift in the baseline term, indicating preserved interaction types but modified thermodynamic driving forces. At the same time, the broader and more polar surface of the morpholinium cation contributes to stronger cation–anion interactions and a more structured liquid environment, in line with the larger positive excess enthalpies derived from IDAC temperature dependence. This modification leads to a higher energetic cost of cavity formation and thus to increased γ_2_^∞^ values and reduced *K*_L,2_ for nonpolar solutes.

Within the regular solution framework, these changes manifest only as a small decrease in the effective Hildebrand solubility parameter, highlighting the limited ability of RST to capture the redistribution of specific and directional interactions revealed by the σ-profiles. Overall, the COSMO-RS analysis provides a molecular-level explanation for the experimentally observed trends, linking increased polarity heterogeneity and stronger cohesive interactions in the morpholinium IL to reduced solvation affinity, particularly for nonpolar solutes.

##### Overall Predictive Performance

The parity plots in Figure 6 compare COSMO-RS predictions of γ_2_^∞^ with experimental values for the pyrrolidinium- and morpholinium-based ILs. The full comparison data set is given in the Supporting Information (Sheet S8). For both systems, the BP-SVP-AM1-COSMO level reproduces the overall trend across several orders of magnitude, with moderate scatter that increases with γ_2_^∞^, particularly in the intermediate and high ranges. The BP-TZVP-COSMO parametrization yields a more compact distribution, especially in the range of γ_2_^∞^ approximately from 1 to 100, indicating improved internal consistency. However, a clear systematic bias is observed, with increasing underestimation of experimental values as γ_2_^∞^ increases. This effect is evident for both ILs and is particularly pronounced for nonpolar solutes. In contrast, the BP-TZVPD-FINE parametrization leads to a strong and systematic overestimation of γ_2_^∞^, which increases progressively with increasing γ_2_^∞^. Deviations frequently exceed 1 order of magnitude at high values, indicating a pronounced bias rather than random scatter. This behavior is more pronounced for the morpholinium-based IL, where predicted values diverge more steeply from the parity line over the entire high-γ_2_^∞^ range.

**Figure 6:**
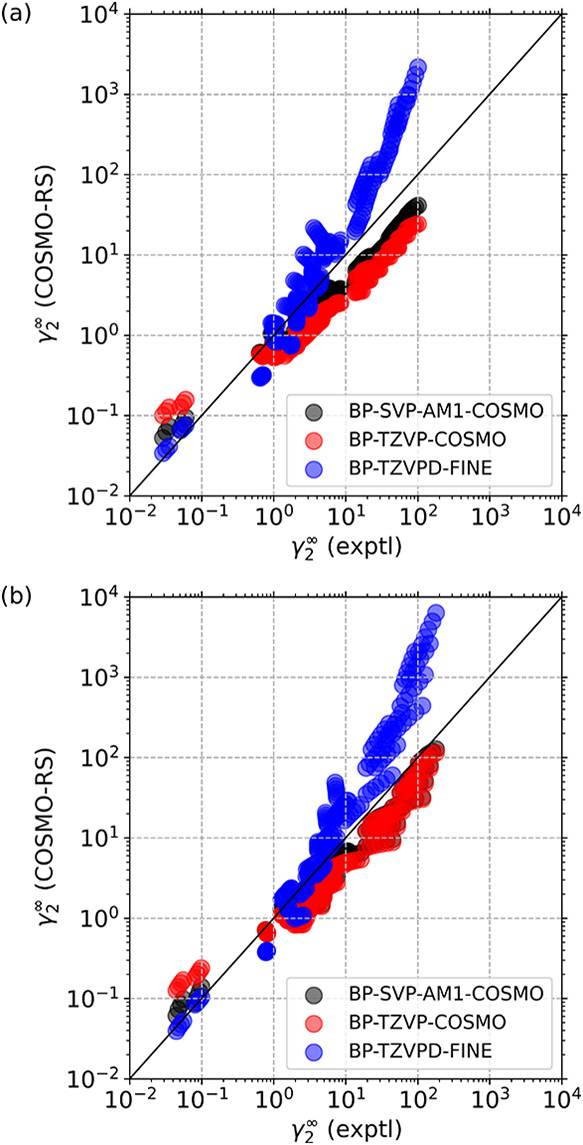
Parity plots comparing experimental and COSMO-RS-predicted infinite dilution activity coefficients (γ_2_^∞^) over the investigated temperature range: (a) 1-ethyl-1-methylpyrrolidinium diethyl phosphate; (b) 4-ethyl-4-methylmorpholinium diethyl phosphate. Results are shown for three levels of theory. The solid line represents perfect agreement.

A comparison between Figure 6a and 6b shows that the morpholinium system exhibits larger systematic deviations for all parametrizations, particularly at high γ_2_^∞^. This indicates that COSMO-RS is more sensitive to inaccuracies in describing the balance of intermolecular interactions in this system, where small errors in predicted interaction energies translate into large relative deviations in activity coefficients.

Overall, none of the parametrizations provides quantitative agreement across the full range of γ_2_^∞^. Increasing the level of theory does not systematically improve predictive accuracy but instead shifts the nature of the error—from moderate scatter (BP-SVP-AM1-COSMO), through systematic underestimation (BP-TZVP-COSMO), to pronounced overestimation (BP-TZVPD-FINE). This behavior indicates that the dominant source of error arises from limitations in the underlying physical description of intermolecular interactions rather than from the level of quantum-chemical detail used to generate the COSMO surface. The magnitude of the deviations increases with γ_2_^∞^, indicating that the largest errors occur for systems dominated by unfavorable solvation and large free-energy penalties.

Among the three tested parametrizations, BP-SVP-AM1-COSMO provides the most balanced overall description of the experimental γ_2_^∞^ data for the studied ILs. Although its predictions exhibit moderate scatter, it does not display the pronounced directional error observed for the higher-level parametrizations. It may therefore be regarded as the preferred COSMO-RS approach for semiquantitative prediction of γ_2_^∞^ in the present systems, while recognizing that significant deviations remain for strongly nonideal cases.

##### Statistical Evaluation of Predictive Accuracy

The predictive performance of COSMO-RS was quantified using the mean absolute percentage error (MAPE) and the root-mean-squared error (RMSE) expressed in logarithmic units

16a $$\mathrm{MAPE}=\frac{1}{N}\sum_{i=1}^{N}\left|\frac{{\mathrm{\gamma ̂}}_{2,i}^{\infty }}{\gamma_{2,i}^{\infty }}-1\right|\times 100\%$$

16b $$\mathrm{RMSE}={\left[\frac{1}{N}\sum_{i=1}^{N}{\left(\ln\frac{{\mathrm{\gamma ̂}}_{2,i}^{\infty }}{\gamma_{2,i}^{\infty }}\right)}^{2}\right]}^{1/2}$$

where *N* denotes the number of data points, and γ̂_2,*i*_^∞^ and γ_2,*i*_^∞^ are the predicted and experimental IDAC values, respectively. For the pyrrolidinium-based IL, the MAPE values are 45.6%, 65.5%, and 213.2% for BP-SVP-AM1-COSMO, BP-TZVP-COSMO, and BP-TZVPD-FINE, respectively, with corresponding RMSE values of 0.694, 1.048, and 1.101. For the morpholinium-based IL, the corresponding errors are 39.4%, 52.3%, and301.6% (MAPE) and 0.604, 0.745, and 1.254 (RMSE). These results show that the overall quantitative accuracy of COSMO-RS for the studied systems is limited and that increasing the nominal level of theory does not lead to systematic improvement. Among the tested parametrizations, BP-SVP-AM1-COSMO provides the most balanced overall performance for both ILs, combining the lowest global error with the smallest systematic bias.

More detailed analysis reveals that the predictive accuracy depends strongly on solute type. BP-SVP-AM1-COSMO provides the most uniform performance across the full set of compounds, with MAPE values for most solutes typically in the range of about (40 to 70)%, although notably lower errors are obtained for selected heterocyclic and strongly polar solutes such as pyridine, thiophene, and acetonitrile. BP-TZVP-COSMO generally increases the errors relative to BP-SVP-AM1-COSMO and is particularly unfavorable for alcohols, for which MAPE reaches 205% in the pyrrolidinium IL and 165% in the morpholinium IL. In contrast, BP-TZVPD-FINE exhibits strongly nonuniform behavior. For several polar solutes, particularly alcohols, ketones, tetrahydrofuran, and 1,4-dioxane, the errors are markedly reduced and in some cases become very small. At the family level, the MAPE decreases to 22.3% and 6.4% for alcohols and to 22.8% and 28.1% for ketones in the pyrrolidinium- and morpholinium-based ILs, respectively. Likewise, tetrahydrofuran is reproduced with MAPE values of only 4.2% and 8.0%. However, this improvement is offset by extremely large errors for dispersion-dominated solutes, especially *n*-alkanes and long-chain alkenes, for which BP-TZVPD-FINE strongly overestimates γ_2_^∞^. At the family level, the corresponding MAPE reaches 826% and 1258% for alkanes, 308% and 478% for alkenes, and 329% and 403% for cycloalkanes in the pyrrolidinium and morpholinium ILs, respectively; for individual solutes, the deviations become extreme, reaching 1500% for *n*-decane in the pyrrolidinium IL and 2600% for *n*-decane in the morpholinium IL. Thus, the error analysis confirms a clear trade-off between the description of polar and nonpolar solutes. BP-SVP-AM1-COSMO performs best for hydrocarbons and other dispersion-dominated systems, whereas BP-TZVPD-FINE can substantially improve predictions for polar solutes but at the cost of catastrophic errors for nonpolar compounds. BP-TZVP-COSMO occupies an intermediate position but does not offer a favorable balance of errors for either class. These results reinforce the conclusion that BP-SVP-AM1-COSMO is the preferred parametrization for semiquantitative prediction over the chemically diverse set of systems investigated here.

The distribution of prediction errors across different solute families is summarized in Figure 7, where box plots of δ ln γ_2_^∞^ = ln γ̂_2_^∞^ – ln γ_2_^∞^ are shown for each COSMO-RS parametrization and IL. Several systematic trends are immediately apparent. For BP-SVP-AM1-COSMO (Figure 7a), the errors are relatively narrowly distributed and predominantly negative for nonpolar solutes such as alkanes, cycloalkanes, and alkenes, indicating a consistent underestimation of γ_2_^∞^. In contrast, polar solutes, particularly alcohols, exhibit positive deviations, reflecting overestimation of solute–IL interactions. Nevertheless, the overall spread remains moderate across all families, confirming the comparatively balanced performance of this parametrization. For BP-TZVP-COSMO (Figure 7b), the same qualitative trends persist but with increased dispersion and more pronounced systematic bias, especially for alcohols, for which large positive deviations are observed. The deterioration in performance relative to BP-SVP-AM1-COSMO is thus associated with both increased variance and stronger family dependent bias. The behavior of BP-TZVPD-FINE (Figure 7c) is markedly different. While the errors for polar solutes, including alcohols and ketones, are substantially reduced and often centered close to zero, indicating improved quantitative agreement, the distributions for nonpolar hydrocarbons are shifted strongly toward large positive values with very broad spreads. This reflects severe overestimation of γ_2_^∞^ and directly corresponds to the extreme MAPE values observed for these families. Overall, Figure 7 clearly illustrates the fundamental trade-off inherent in the COSMO-RS parametrizations: improvements in the description of polar interactions are achieved at the expense of dispersion-dominated systems, whereas BP-SVP-AM1-COSMO provides the most consistent compromise across the full range of solute types.

**Figure 7:**
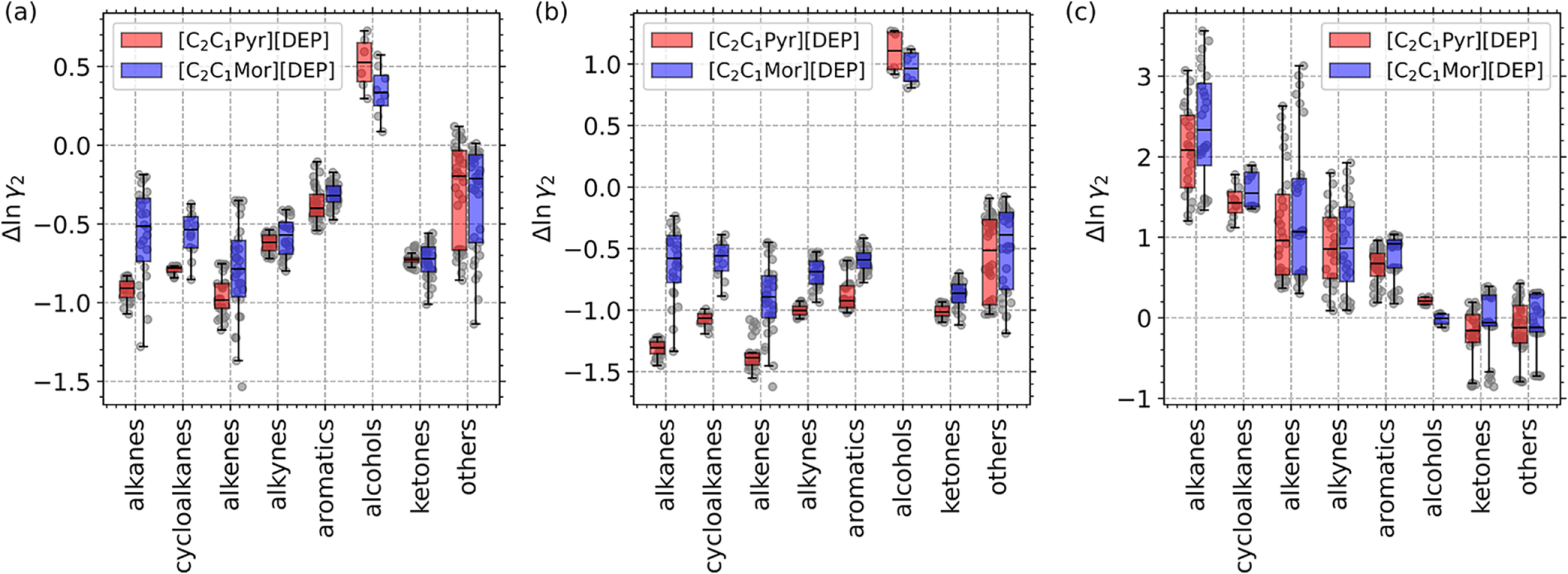
Distribution of prediction errors Δ ln γ_2_^∞^ = ln γ̂_2_^∞^ – ln γ_2_^∞^ for COSMO-RS grouped by solute family: (a) BP-SVP-AM1-COSMO; (b) BP-TZVP-COSMO; (c) BP-TZVPD-FINE. Results are shown for both ILs over the investigated temperature range. Boxes indicate interquartile ranges with medians, whiskers show data ranges, and individual points are plotted in gray. The horizontal line represents zero deviation.

##### Qualitative Predictive Capability

Given the limited quantitative accuracy, the qualitative performance of COSMO-RS was evaluated using Spearman’s rank correlation coefficients. For both ILs, ρ exceeds 0.96 across all temperatures, reaching values up to 0.99. The coefficients increase slightly with temperature and are very similar for all parametrizations, indicating that COSMO-RS reliably preserves the relative ordering of solute affinities even when absolute errors are large. Notably, even BP-TZVPD-FINE retains high rank correlation despite substantial systematic deviations, confirming that its errors are primarily systematic rather than random.

The ability to predict the effect of cation substitution was evaluated by comparing the sign of the difference in γ_2_^∞^ between the morpholinium- and pyrrolidinium-based ILs. All parametrizations achieve 100% accuracy, correctly reproducing the direction of this effect for all data points, demonstrating that COSMO-RS consistently captures the relative change in solvation upon cation functionalization.

Temperature dependence was assessed via the sign of the slope in ln γ_2_^∞^ versus 1/*T*. For the pyrrolidinium-based IL, 25, 27, and 23 out of 34 slopes are correctly predicted for BP-SVP-AM1-COSMO, BP-TZVP-COSMO, and BP-TZVPD-FINE, respectively. The discrepancies are concentrated for aromatics (benzene, toluene), heterocycles (thiophene, pyridine), nitriles (acetonitrile), ketones, and 1-nitropropane, indicating that COSMO-RS has difficulty reproducing subtle enthalpy–entropy balances for systems with competing interaction types. For the morpholinium-based IL, agreement improves to 29, 30, and 32 correct predictions, with disagreements largely limited to alkynes and selected polar solutes such as tetrahydrofuran, acetonitrile, and 1,4-dioxane. This indicates a more consistent description of temperature effects in the morpholinium system.

Analysis of the intercept term (corresponding to the entropy contribution) reveals significantly lower predictive accuracy. While slope agreement exceeds (70 to 90)% in most cases, intercept agreement is markedly poorer, particularly for the morpholinium IL, where fewer than half of the intercepts are correctly reproduced for BP-SVP-AM1-COSMO and BP-TZVP-COSMO. This indicates that COSMO-RS captures enthalpic trends more reliably than entropic contributions, consistent with the TD-LSER analysis showing dominant temperature-dependent (enthalpic) terms.

Overall, COSMO-RS provides a robust qualitative description of solute–IL interactions. It reliably captures relative solute affinities, correctly predicts the direction of cation substitution effects, and reproduces temperature trends with good accuracy, particularly for the morpholinium-based IL. However, the significantly lower accuracy in intercept prediction and the systematic errors observed for certain solute classes indicate that the model struggles to accurately describe the balance of enthalpic and entropic contributions to solvation.

##### **σ**-Profile-Derived Descriptor Correlations

While COSMO-RS provides direct predictions of γ_2_^∞^, its quantitative accuracy for the present systems is limited and depends strongly on the chosen parametrization. The descriptor-based correlations developed here therefore serve a complementary purpose. By relating experimental IDACs directly to COSMO-derived σ-profile features, they provide a transparent and physically interpretable mapping between molecular surface polarity characteristics and solvation thermodynamics. In addition, this approach enables partial decoupling of systematic errors inherent to COSMO-RS from the underlying molecular descriptors, allowing assessment of whether the experimental trends can be rationalized within the COSMO framework itself. Accordingly, a regression analysis based on COSMO-derived σ-profile descriptors was performed to quantify these relationships. The corresponding design matrix and regression statistics are provided in the Supporting Information (Sheets S9 and S10, respectively).

Among the considered size descriptors, the COSMO surface area (*A*_COSMO,2_) provided slightly better statistical performance than volume (*V*_COSMO,2_) and was therefore retained in the final models.

Analysis of the descriptor space defined in eq 10 reveals consistent and physically meaningful trends for both ILs that closely parallel the interaction patterns identified previously from IDAC, LSER, and RST analyses. Nonpolar hydrocarbons are characterized by narrow σ-profiles (small *w*_σ,2_) and negligible hydrogen-bonding surface fractions, indicating that their solvation is governed primarily by cavity formation effects. This is reflected in the systematic increase of γ_2_^∞^ with increasing *A*_COSMO,2_, consistent with the dominant role of dispersive interactions and the free-energy cost of accommodating large apolar molecules within the IL structure. In contrast, unsaturated and aromatic solutes exhibit broader σ-profiles and moderately negative values of the electrostatic matching parameter Π_2_, indicating enhanced complementarity with the ionic environment and correspondingly lower activity coefficients. Strongly polar solutes, including alcohols, ketones, nitriles, and heterocycles, display the largest values of *w*_σ,2_ together with significant hydrogen-bond donor and acceptor surface fractions, leading to pronounced stabilization through specific and electrostatic interactions and, consequently, to markedly reduced γ_2_^∞^ values. Overall, the COSMO-derived descriptors capture the continuous transition from size-controlled solvation of nonpolar solutes to interaction-dominated behavior for polar compounds.

The final correlations obtained at *T* = 338.15 K are given by

17 $$\ln\gamma_{2}^{\infty }=4.558\left(0.610\right)-13.381\left(1.132\right)w_{\sigma ,2}/e\cdot {\mathrm{nm}}^{-2}+21.852\left(2.493\right)A_{\mathrm{HBD},2}-15.118\left(2.164\right)A_{\mathrm{HBA},2}-2.383\left(0.407\right)\Pi_{2}+0.0158\left(0.002\right)A_{\mathrm{COSMO},2}/{Å}^{2}$$

for the [C_2_C_1_Pyr]­[DEP] IL, and

18 $$\ln\gamma_{2}^{\infty }=5.055\left(0.654\right)-12.964\left(1.141\right)w_{\sigma ,2}/e\cdot {\mathrm{nm}}^{-2}+16.858\left(2.202\right)A_{\mathrm{HBD},2}-11.363\left(2.282\right)A_{\mathrm{HBA},2}-2.094\left(0.398\right)\Pi_{2}+0.0157\left(0.002\right)A_{\mathrm{COSMO},2}/{Å}^{2}$$

for the [C_2_C_1_Mor]­[DEP] IL. In both cases, the statistical quality of the regression is excellent (*N* = 34, *R* ^2^ = 0.984, adjusted *R* ^2^ = 0.982 and 0.981, *F* > 330), and all coefficients are highly significant (*p* < 0.001), confirming that the descriptor set provides a robust quantitative representation of the experimental IDACs. It should be noted that the σ-profile descriptors employed in these correlations were derived from COSMO calculations performed at the BP-SVP-AM1-COSMO level. This choice is consistent with the predictive performance analysis presented above, where this parametrization provided the most balanced description of γ_2_^∞^ across the full range of solute types. In particular, higher-level parametrizations were found to introduce systematic biases, most notably a strong overestimation of activity coefficients for nonpolar solutes, which would translate into distorted surface descriptors and reduced transferability in regression analysis. The BP-SVP-AM1-COSMO level therefore provides a more balanced representation of molecular surface polarity, ensuring consistency between COSMO-RS predictions and the descriptor-based correlations.

Interpretation of the regression coefficients shows that solvation thermodynamics in both ILs is governed primarily by surface polarity heterogeneity and hydrogen-bonding capability, with an additional systematic contribution arising from molecular size. The large negative coefficient associated with *w*_σ,2_ indicates that solutes with highly nonuniform surface charge distributions are strongly stabilized in the IL environment due to their ability to engage simultaneously in dispersive, electrostatic, and hydrogen-bonding interactions. Hydrogen-bonding surface fractions also exert a pronounced influence: the positive coefficient for *A*_HBD,2_ and negative coefficient for *A*_HBA,2_ reflect the asymmetric nature of solute–IL interactions, where electron-rich regions of the solute are preferentially stabilized. The negative contribution of the electrostatic matching parameter Π_2_ further highlights the importance of charge complementarity with the IL in reducing IDACs. Finally, the positive coefficient of *A*_COSMO,2_ confirms the increasing energetic penalty associated with cavity formation as solute size increases.

A direct comparison of the two regressions provides additional insight into the effect of cation functionalization. While the overall structure of the correlation remains unchanged, all interaction-related coefficients (*A*_HBD,2_, *A*_HBA,2_, and Π_2_) are systematically smaller in magnitude for the morpholinium-based IL. This suggests a reduced apparent sensitivity of ln γ_2_^∞^ to specific and electrostatic interactions within the present descriptor space, fully consistent with the LSER results showing nearly unchanged selectivity but reduced overall solvent affinity, as well as with the experimentally observed increase in γ_2_^∞^. In contrast, the coefficient associated with molecular size remains essentially unchanged, demonstrating that cavity formation effects are not significantly affected by the cation modification. These results show that insertion of the oxygen atom into the cation ring primarily weakens solute–IL interaction strength rather than altering the fundamental balance between dispersive and steric contributions.

It should be noted that the relatively large condition numbers of the regressions indicate partial multicollinearity among the descriptors, which is inherent to their common origin from σ-profile integrals. Consequently, the coefficients should be interpreted as conditional contributions within a multivariate descriptor space rather than as independent interaction parameters. Nonetheless, the consistency of trends across both ILs and their clear physical interpretation confirm the robustness of the obtained correlations.

## Conclusions

The experimental IDAC data demonstrate that solvation in the studied ILs is governed by a balance between liquid-state cohesion and specific solute–IL interactions. Nonpolar solutes exhibit large, size-dependent IDAC values dominated by the energetic cost of cavity formation, whereas polar solutes show much lower values due to favorable dipolar and hydrogen-bonding interactions. Temperature-dependent analysis confirms that nonpolar solvation is both enthalpically and entropically unfavorable, while polar solutes are stabilized primarily by favorable enthalpic contributions. Cation functionalization leads to a systematic increase in IDAC, particularly for nonpolar solutes, indicating a higher thermodynamic cost of solvation in the morpholinium system. This effect diminishes with increasing solute polarity, demonstrating that cation modification primarily shifts the baseline thermodynamic cost of solvation rather than altering the hierarchy of solute–IL interactions. The consistency of these trends with literature data for related pyrrolidinium- and morpholinium-based ILs further suggests that the observed behavior reflects a general consequence of oxygen incorporation into the cation ring rather than a system-specific effect.

Modeling results support this interpretation. LSER analysis shows that replacing the pyrrolidinium core with morpholinium produces an overall decrease in solvent affinity while preserving the relative contributions of dispersion, polarity, and hydrogen-bonding interactions. Regular-solution analysis indicates only minor differences in the effective Hildebrand solubility parameter, highlighting the limited ability of a single scalar descriptor to capture the observed changes. COSMO-RS provides a reliable qualitative description, correctly reproducing relative solute affinities, the direction of the cation effect, and most temperature trends, but lacks quantitative predictive accuracy. Collectively, the modeling shows that insertion of oxygen into the cation ring shifts the thermodynamic baseline of solvation while preserving the interaction pattern. In practical terms, the ring-oxygen substitution makes the IL less accommodating toward cavity formation while leaving the relative ordering of solute interactions unchanged.

These findings show that targeted cation functionalization can provide a rational route to tuning solvation thermodynamics in ILs and highlight the sensitivity of phosphate-based systems to controlled modification of liquid-state interaction balance.

## Supplementary Material



## Notes

Gas–liquid partition coefficients calculated from experimental IDACs; LSER and TD-LSER design matrices and regression statistics; data used for determination of Hildebrand solubility parameters; COSMO σ-profiles of ionic-liquid ions and molecular solutes for all quantum-chemical parametrization levels; comparison of experimental and COSMO-RS-predicted IDACs; and COSMO descriptor design matrix and regression statistics (XLSX)
